# Factors Influencing the Uptake of Seasonal Influenza Vaccination among Community-Dwelling Older Adults during COVID-19: A Mixed Methods Study

**DOI:** 10.3390/vaccines11030641

**Published:** 2023-03-13

**Authors:** Yi Xu, Min Ting Alicia See, Fazila Aloweni, Chun Hui Fion Koh, Cheng Gaik Irene Tan, Xiao Hui Xin, Wee Hoe Gan, Lian Leng Low, Su Fee Lim

**Affiliations:** 1 Community Nursing, Population Health and Integrated Care Office (PHICO), Singapore General Hospital, Singapore 169608, Singaporeirene.tan.c.g@sgh.com.sg (C.G.I.T.);; 2 Nursing Division (Nursing Research), Singapore General Hospital, Singapore 169608, Singapore; 3 Health Services Research Unit, Singapore 169608, Singapore; 4 Occupational and Environmental Medicine, Singapore General Hospital, Singapore 169608, Singapore; 5 Executive Office, SingHealth Community Hospitals, Singapore 168582, Singapore; 6 Population Health and Integrated Care Office (PHICO), Singapore General Hospital, Singapore 169608, Singapore; 7 Outram Community Hospital, SingHealth Community Hospitals, Singapore 168582, Singapore

**Keywords:** older adult, influenza, vaccine, vaccination, barrier, enabler, mixed methods

## Abstract

**Background**: Despite making the influenza vaccine accessible and affordable, vaccination rates remained low among community-dwelling older adults. Therefore, this study aimed to explore the factors influencing vaccine uptake and the impact of COVID-19 on vaccine uptake among community-dwelling older adults in Singapore. **Methods:** A mixed methods study involving a survey and semi-structured interviews were conducted between September 2020 and July 2021. Community-dwelling older adults aged ≥ 65 years were recruited from 27 Community Nurse Posts. Data on participants’ demographics, health condition(s), vaccination status, attitudes towards influenza infections and vaccinations, willingness to pay, intention for future vaccination and source of information were collected via the survey. Semi-structured interviews were conducted to understand vaccination experiences, key enablers and barriers, and the impact of COVID-19 on vaccine uptake. All interviews were analysed using Braun and Clarke’s thematic analysis. Quantitative data were analysed using descriptive statistics, chi-square tests and multinomial logistic regressions. **Results:** A total of 235 participants completed the survey. Living arrangement was a statistically significant contributing factor for influenza vaccine uptake (ꭓ^2^= −0.139; *p* = 0.03). Participants who lived alone were 2.5 times more likely to be vaccinated than those living with others (OR = 2.504, 95% CI: 1.294–4.842, *p* = 0.006). Avoidance of getting infected (82.5%), avoidance of transmission to others (84.7%), and advice from healthcare professionals to receive vaccination (83.4%) were key enablers, while concerns about possible side effects (41.2%), the effectiveness of the vaccine (42.6%), and not having enough information (48.1%) were barriers. Twenty participants were interviewed. The findings were congruent with the survey results. Five themes were identified as follows: (1) Perceived importance of influenza vaccination, (2) Sphere of influence, (3) Healthcare schemes and medical subsidies, (4) Psychological impediments, and (5) Inconsistent emphases at various touch points. **Conclusions:** Greater public health efforts are needed to reach out to the larger population of older adults of different living arrangements and those concerned about the possible side effects and effectiveness of the influenza vaccine. Healthcare professionals need to provide more information to address these concerns, especially during COVID-19, to encourage vaccine uptake.

## 1. Background 

Influenza, a contagious viral respiratory infection that commonly affects people of all age groups, remains one of the most serious diseases affecting public health, leading to increased morbidities, mortalities, and hospitalisations worldwide [1,2,3]. The World Health Organization (WHO) has estimated 3 to 5 million cases of severe influenza illness, with about 290,000 to 650,000 deaths every year globally [4]. Older adults aged 65 years and above, especially those with chronic diseases, face a higher risk of influenza and related complications, which makes them a priority group for vaccination [5]. An estimated 90% of influenza-associated deaths were reported in older adults aged 65 years and above [6]. Amongst them, those aged 75 years and above were 47 times more likely to be hospitalised for influenza than younger adults [7]. 

The influenza vaccination (InVa) has been recommended as an evidence-based and cost-effective health intervention in preventing influenza infection, transmission to others, related complications and deaths [3,8]. InVa has averted substantial complications, including 58,000 hospitalisations, 2.3 million outpatient visits and 3500 deaths during the 2018–2019 influenza in the United States [9]. For persons infected, InVa resulted in less severe symptoms and reduced doctor visits [10,11,12]. Although the economic burden that is associated with influenza remains unknown in Asia [13], national vaccination strategies targeting the elderly and other at-risk groups could result in savings of SGD 36 million per 100,000 population over ten years [14]. 

Influenza is known to circulate all-year round and typically has two peak periods (May to July; November to January) [15]. The Singapore MOH Expert Committee for Immunization has since recommended that InVa is administered to populations at greater risk of influenza and its related complications, such as those aged 65 years and above and those with chronic diseases [16]. The National Adult Immunization Schedule was, thus, set up to provide vaccination guidance [15]. However, unlike other vaccinations, which are required only once or twice in a lifetime, InVa needs to be conducted at least once a year for effective vaccination. This annual regimen may be onerous for some. In 2017, a national survey conducted in Singapore reported the InVa rate was only 14% [15]. As such, efforts were made to improve its affordability and accessibility. Yet, the InVa rate remained consistently low [17]. In light of this, studies have been conducted internationally [3,17,18,19] and also in local contexts [20,21,22] to identify the factors affecting uptake. Older age [17,18,21] and having at least one chronic condition [18,19,21] were positively associated with InVa. Key enablers identified included protection from influenza [3,17], advice from healthcare professionals [20,21,22], as well as reminders and support from family and friends [3,22]. Concerns about vaccine side effects [3,17,19,22], beliefs in own immunity and perceived low risk [17,19,20,22] were key barriers. Most of the studies were conducted using a single research design which might not yield a comprehensive understanding compared to a mixed methods approach. The studies were also conducted prior to COVID-19, and as such, the impact of COVID-19 on vaccine uptake among community-dwelling older adults is unknown. 

Community nurses promote preventive health through health risk assessment and screening, health education and counselling, community mobilisation, care coordination, case management, and monitoring and rehabilitation. Promoting InVa uptake among older adults is considered one of the key target components. As such, it is essential to identify the factors influencing InVa uptake among our community-dwelling older adults to achieve a comprehensive understanding of the specific enablers and barriers and to support the design of effective interventions that could both increase and sustain InVa uptake in the long term. 

## 2. Aims 

This study aimed to explore the factors influencing InVa uptake and the impact of COVID-19 on vaccine uptake among community-dwelling older adults in Singapore. 

## 3. Methods 

### 3.1. Design and Setting 

A mixed methods study involving a one-time survey and face-to-face semi-structured interviews were conducted across five Communities of Care (CoCs), namely Bukit Merah, Telok Blangah, Tiong Bahru, Chinatown, and Katong, under the Singapore General Hospital Community Nursing service boundary in the Southeast region of Singapore. 

### 3.2. Participant Recruitment 

Community-dwelling older adults aged 65 years and above and able to read, write and converse in English were recruited from 27 Community Nurse Posts (CNPs) across the five identified CoCs. Based on the sample size calculation and InVa rate of 14% [15], we estimated that a sample size of 196 participants would be adequate. However, after factoring in a 20% dropout rate, such as incomplete responses, the final sample size was 235 participants. Stratified sampling was used to recruit 47 participants from each of the CoC from a daily list of resident appointments. Individuals were screened to assess their eligibility and willingness to participate. Those who were cognitively impaired were excluded. 

### 3.3. Data Collection Instruments 

The multicomponent survey questionnaire was designed based on adaptations from local studies on influenza in community-dwelling older adults [20,21,23] and primarily grounded in the Health Belief Model [23]. The survey was pilot-tested and reviewed by study members who were experienced practitioners in community nursing, family medicine and population health. The finalised survey contained: (i) demographics including health condition(s) and vaccination status, (ii) attitudes towards influenza infections and vaccinations, using a 5-point Likert scale from ‘Strongly agree’ to ‘Strongly disagree’, (iii) willingness-to-pay for vaccination, using a 3-point Likert scale from ‘Very willing’ to ‘Not willing’ to detect for changes in willingness to pay with and without financial subsidies, (iv) intention for future vaccination, using a 5-point Likert scale from ‘Very important’ to ‘Not important at all’, and (v) source of information. 

The semi-structured interview guide was developed based on two studies [22,24], with reference to the Health Belief Model and Theory of Reasoned Action [24]. The interview guide aimed to explore the participants’ InVa experiences and factors that could motivate or discourage them from vaccine uptake. In addition, the participants were asked about the best approach and healthcare support to encourage InVa uptake in the community. Finally, a question was posed to them to explore how COVID-19 has impacted their decision to be vaccinated against influenza. 

The Interview questions were:

1. What are the factors that motivate or discourage you to uptake InVa?
2. What do you think is the best approach to encourage yearly InVa uptake in the community?
3. What other healthcare support is needed to increase InVa uptake in the community?
4. Has COVID-19 influenced your decision to be vaccinated against influenza?

### 3.4. Data Collection Procedures 

Upon successful recruitment, the participants were asked to complete the survey questionnaire. Thereafter, their vaccination status was verified from their medical records to prevent recall bias. Subsequent interviews were conducted with 20 survey respondents who agreed to be interviewed using the semi-structured interview guide. Due to the need for safe distancing and scaling down of group activities during COVID-19, the researchers were not able to conduct the focus groups as planned. Instead, eight individual face-to-face interviews and twelve telephone interviews were conducted between January to March 2021 and May to July 2021, respectively. Data saturation was considered achieved at the 20th telephone interview, where no additional data were attained, after which data collection ceased [25]. All interviews were audio-recorded and transcribed verbatim for data analysis. 

### 3.5. Data Analysis 

The Statistical Package for Social Sciences Version 25 was used to analyse the quantitative data. Descriptive statistics were used to report the participants’ demographics. Chi-square tests (ꭓ^2^) were performed to test for associations between the participants’ demographics and InVa uptake. Multinomial logistic regressions were then used to identify the factors that influenced InVa uptake between the vaccinated, non-vaccinated, and unsure groups [26]. *p* values ≤ 0.05 were considered statistically significant. 

The interview transcripts were checked for accuracy before the qualitative data were analysed using Braun and Clarke’s [27] thematic analysis. Three researchers, XY, SMTA and FKCH from the study team independently read the transcripts several times to immerse fully in the data before they extracted the keywords and phrases of interest. The researchers then met up to compare their notes and discuss the potential themes and subthemes. All disagreements were resolved through open discussion until a consensus was reached. The data collection and analysis process was concurrent and iterative until data saturation was achieved. 

### 3.6. Ethical Considerations 

Ethics approval was obtained from SingHealth Centralized Institutional Review Board (CIRB Ref: 2020/2305). All participants were informed of the study objectives and procedures in length before written informed consent was obtained. 

## 4. Results 

### 4.1. Demographics 

A total of 235 participants were recruited. The majority were female (57.0%), Chinese (68.5%), and aged 75 years and above (60.9%). Most participants lived with others (66.8%) in either a one or two-room Housing and Development Board flat (Singapore governmental housing) (70.2%) and were currently not working (91.9%). Approximately two-thirds had no formal education or only primary education (70.6%). Half of the participants (50.6%) had two or more comorbidities, e.g., diabetes mellitus, cancer, and a heart condition. Most participants indicated that they had a regular family doctor or polyclinic that they returned to for medical treatments (94%) and lived near the primary care facilities where InVa was offered (96.6%). Of the participants surveyed, 39.6% (*n* = 93) had received an InVa in the past year. Among those who were unvaccinated, 24.8% had not heard of InVa (Table 1). 

### 4.2. Quantitative Survey Results

#### 4.2.1. Living Arrangement as an Associated Factor for InVa Uptake

Among the demographics, living arrangement was the only associated factor for InVa uptake in the past year (ꭓ^2^ = −0.139, *p* = 0.03) (Table 1). Participants who lived alone were 2.5 times more likely to be vaccinated than those living with others (OR = 2.504, 95% CI: 1.294–4.842, *p* = 0.006) (Table 2).

#### 4.2.2. Attitudes towards Influenza Infections and Vaccinations

Of the 235 participants surveyed, 72.7% agreed that influenza is a potentially serious illness, and 73.6% considered InVa important to protect against infection. Participants identified the groups requiring regular vaccination as adults aged ≥ 65 years (71.9%) and those with chronic diseases (71.5%). Less than half (47.2%) perceived they were at risk of infection. Among the participants, 61.8% perceived that vaccination was needed only before an overseas trip (Table 3).

#### 4.2.3. Intention for Future Vaccination

From the survey results, the majority of participants had indicated avoidance of getting infected (82.5%), avoidance of transmission to others (84.7%), and advice from healthcare professionals to receive vaccination (83.4%) as key enablers influencing intention for future vaccination while concerns about possible side effects (41.2%), the effectiveness of the vaccine (42.6%), and not having enough information (48.1%) were key barriers (Table 4).

#### 4.2.4. Willingness to Pay for Vaccination

Approximately half of the participants (55.8%) expressed willingness to pay for vaccination at SGD 35 per dose. Most participants (75.7%) were, however, only willing to vaccinate if the cost is fully covered under MediSave, a national healthcare savings scheme (Table 5).

#### 4.2.5. Source of Information

The most preferred vaccination site was the polyclinic (55.3%), community spaces (39.1%), and general practitioner/family clinic (21.7%), while the most preferred source of information was the HCPs (72.8%), followed by television/radio (31.1%) (Table 6).

### 4.3. Qualitative Interview Findings

Twenty participants took part in the interviews. Amongst them, only eight had received an InVa in the past year (Table 7). Five main themes and seven subthemes were identified.

### 4.4. Theme 1: Perceived Importance of InVa

The participants perceived that by being vaccinated, they could avoid getting infected with influenza and, at the same time, reduce transmission to others.

*Subtheme 1a: Avoidance of getting infected*

Participants acknowledged the importance of InVa uptake and viewed it as a significant measure to protect themselves against influenza in old age and during COVID-19. One participant further elaborated on how COVID-19 had amplified the importance of InVa and recognised its value in building personal defences against influenza.

“I scared (I) will get infected. Here all old people, then easy to get infection.” (P5)

“It is for prevention, my age is older, it is safer (for me) to receive (InVa).” (P12)

“I need to avoid going out as much as possible to prevent infection, but I will still jab to protect myself.” (P16)

“I wanted to take the vaccination to defend myself. Because of COVID-19, I was worried and felt it is important for me to build my defense… So, I decided to take up the vaccination to protect myself from getting the flu.” (P8)

*Subtheme 1b: Reduce transmission to others*

Besides receiving InVa to prevent the risk of infection, participants also shared that InVa uptake could reduce the risk of transmission to others.

“You must take more strong precautions… don’t let it [influenza] spread… You don’t wait till it comes then you decide to do something. You stop it before it comes.” (P3)

### 4.5. Theme 2: Sphere of Influence

Participants identified that adequate prompters from significant others, such as one’s HCPs, family, peers, and ex-colleagues, influenced their decision-making to vaccinate. Several participants also highlighted the need for community engagement programmes to expand the sphere of influence. 

*Subtheme 2a: Adequate prompters from significant others*

Many participants shared that they decided to get vaccinated based on their HCPs’ recommendation.

“The nurses encouraged me to get it [InVa].” (P3)

“My family physician says… vaccination can build up my lungs stronger, so I follow the doctor’s suggestion.” (P12)

“I was recommended (InVa) by polyclinic doctor and lung specialist.” (P14)

“My cancer doctor recommended me to go for influenza vaccination, so I go.” (P15)

Others mentioned that they were urged by their family members to get vaccinated, and therefore, they decided to vaccinate.

“My granddaughter… she asks me to go [for InVa].” (P10)

For the participants who lived alone and were not aware of InVa, many gained awareness through friends and peers who were vaccinated.

“We based on hearsay to learn and be made aware of such.” (P12)

“Many people asked me to get vaccinated, so I did… my friends have all taken and encouraged me to go.” (P9)

“Last time I didn’t go… but because my friends have injected and gave me the flyer then I understand what is going on.” (P16)

*Subtheme 2b: Community engagement programmes*

Participants suggested that having community engagement programmes, such as health talks and vaccination drives, could raise awareness of InVa and promote its uptake.

“It will be better that Residents’ Committee… come forward to organize some talks for us to raise awareness of the flu problem among many residents.” (P7)

“You can have a nurse come into the [senior activity] centre… encourage them to go and listen to the talk.” (P2)

“Senior Activity Centers can organise events to promote vaccination.” (P13)

However, the accessibility of these community engagement programmes, such as proximity to the participants’ homes, were key considerations as to whether the participants successfully attended the education talks and vaccination drives. Having the transport to ferry the participants to and from the sites would facilitate their travel.

“Have to think [of] the venue… have to travel. So if it’s too far, they are discouraged… [If] there’s a bus for them, bring them safely there… then come back. So maybe that will help.” (P7)

### 4.6. Theme 3: Healthcare Schemes and Medical Subsidies

Participants indicated that the introduction of the national healthcare savings scheme (MediSave) reduced the cost of vaccination and made the vaccination more affordable than it previously used to be. As such, more participants were willing to vaccinate.

“Medical subsidy helped me too.” (P15)

“Because my friend had injected and told me that the price is very cheap and can use my MediSave to deduct, that is why I will go.” (P16)

### 4.7. Theme 4: Psychological Impediments

Several psychological impediments, such as perceived lack of vulnerability, perceived low priority, and fear of side effects, deterred the participants from vaccinating.

*Subtheme 4a: Perceived lack of vulnerability*

Several participants expressed a perceived lack of vulnerability in getting influenza which led them to trivialise the importance of InVa. From the participants’ views, as long as they were healthy, they did not see the need to vaccinate. Others opined that if they did not engage in social activities, go outside of the home or travel overseas, their risk of getting infected was much lower, and hence, there was no need for vaccination.

“Previously taken this jab [InVa] before, but after that, I did not continue. I did not jab but was well throughout, so I assumed I do not need to regularly do it.” (P18)

“I did not go overseas… I didn’t go out… I’m not going out… I don’t interact a lot.” (P11)

“I did not travel overseas. Why should I take this [InVa]? Go overseas has higher chance of getting it, so better to take if I go overseas.” (P17)

“Senior Activity Center is right opposite my block; I rarely go there to join activities.” (P9)

*Subtheme 4b: Perceived low priority*

Participants perceived the need to fulfil their daily needs and necessities were far more crucial than getting vaccinated. Hence, they did not view the urgency to vaccinate and delayed doing so. One participant further shared about her “one jab is enough” mentality, and since she had already received the COVID-19 vaccination, she did not think getting the InVa was necessary.

“They feel that day to day needs are more important than the vaccination.” (P2)

“I hardly have any flu or runny nose… so I do not have the urgency to go for flu jab… Lately, I had COVID-19 vaccination, I do not think influenza vaccination is necessary… enough, one jab is enough.” (P18)

*Subtheme 4c: Fear of side effects*

Participants who were hesitant to receive InVa shared that they were fearful of its side effects. Such concerns were most prevalent among participants of old age, living alone, and those with a medical history of allergies, multiple comorbidities, and immunocompromised status.

“I watched on YouTube, [InVa] have side effects, I’m not prepared for that… At this age [81 years old] … I’m not too confident, that’s why I never did it.” (P3)

“I am living alone. It is very worrisome when I cannot take care of myself if I cannot recover from the side effects.” (P15)

“I have some allergies. Because I had the tetanus vaccination before. So, I had a very serious consequence… So now if I inject, I will be more cautious.” (P7)

“I still wouldn’t go (for vaccination) … Because my body has many problems… I still have many chronic diseases.” (P4)

“I am still on chemotherapy treatment… very worrisome when I cannot take care of myself if I cannot recover from the side effects.” (P15)

“I do not like to take vaccination… I worry about the side effects… I had cancer and have done chemotherapy before… I like to take it naturally [without InVa].” (P19)

Following media reports on the negative side effects of InVa and its temporary suspension in another country, one participant further voiced out his fear of vaccinating based on the news.

“It [InVa] was supposed to be very good… they found out that it got side effects, then they stopped it, and then they started again… I’m afraid.” (P3)

### 4.8. Theme 5: Inconsistent Emphases at Various Touch Points

While some participants had received adequate prompters from significant others, others did not. Further exploration with these participants revealed that they did not receive any information or medical recommendation to receive InVa in the community or healthcare settings.

“I did not receive any information from Community Center or Resident Committee.” (P9)

“I followed up [in the clinic] for diabetes… but I did not receive any information about the flu vaccination before.” (P10)

“[Doctors and nurses] did not recommend me this [InVa].” (P17)

## 5. Discussion

Seasonal influenza prevails as a public health threat, in which the most effective way of preventing its infection, transmission, associated complications and deaths is through regular vaccinations. Yet, vaccination rates have remained relatively low among our community-dwelling older adults. This spurred us to conduct the study to explore and identify the factors influencing vaccine uptake and the impact of COVID-19 on vaccine uptake among community-dwelling older adults in Singapore.

Living arrangement was identified to be significantly associated with InVa uptake. Our study showed that participants living alone were more likely to be vaccinated than those living with others. This finding contrasted the systematic review and meta-analysis of the social determinants of InVa uptake that individuals living alone are associated with lower vaccination rates [16]. Our finding also opposed the study in the United States, where participants who lived alone were 0.42 times less likely to vaccinate than those who lived with others [28]. One possible reason for this discrepancy could be the older adults who lived alone in our study were already recruited under our community nursing programme. Therefore, despite difficulties in managing their health conditions and the lack of conjugal support in health-related matters, these older adults received follow-ups and visits from community nurses who would track their vaccination statuses and, in turn, provide targeted health coaching on preventive health, including InVa [29]. In addition, these older adults were also being followed up closely by various community agencies for social connectedness due to their current living arrangements.

A sphere of influence emerged as a double-edged sword that could either motivate or discourage our participants from vaccinating. Beginning at the core of the sphere of influence, the importance of the family unit in decision-making is exemplified in our interview findings, where having dialogues with family members exposed our participants to more opportunities to obtain vaccine information and an invitation to take the vaccination. Similar results were noted in two other studies that those living with family members who were vaccinated and had no thoughts of getting vaccinated soon took the vaccination [30,31]. Extending from the family unit to the friend and peer zone, our participants who lived alone shared that they gained awareness of the importance of vaccination from friends and peers who were vaccinated. Further exploration with our participants revealed that HCPs were key influencers in their decision-making to vaccinate. Studies also showed that regular visits with HCPs were associated with increased opportunities for HCPs to recommend InVa uptake [24,32,33]. Our findings, however, revealed that while some participants had received adequate prompters from their HCPs to get vaccinated, others did not. The lack of personal recommendations from one’s HCPs to uptake InVa could have reinforced our participants’ impression that the vaccination was unnecessary and influenced their decision to not vaccinate [22,31].

The Health Belief Model is a social psychological health behaviour change model that is used to explain and predict health-related behaviours, particularly in regard to the uptake of health services. The model posits that a person’s belief in a personal threat of an illness or disease, together with the person’s belief in the effectiveness of the recommended health behaviour or action, will predict the likelihood the person will adopt the health behaviour [34]. Our study identified avoidance of getting infected as a contributory factor towards intention for future vaccination. However, although most of our participants acknowledged the importance of InVa and viewed its uptake as a preventive measure to gain immunological protection, less than half had perceived they were at risk of influenza and required InVa. This finding relates to a preceding study which examined the predictors of vaccine uptake intentions for influenza. According to the study, individuals who calculated their risk of getting infected as low were less likely to have the intention to vaccinate and get vaccinated [23]. Self-perceived good health and having a strong immune system were cited as common reasons for participants to postpone or refuse influenza vaccination [17,19,24]. Nevertheless, given the current COVID-19 situation, the importance of InVa to building immunity has been amplified in our study, possibly to minimise the impact of having two serious respiratory viruses circulating at once.

Besides receiving InVa to reduce the risk of infection, our participants also shared that one of the major motivators for InVa uptake was to reduce the risk of transmission to others. The participants recognised that being unvaccinated, they could remain as asymptomatic carriers and unknowingly transmit the virus to close contacts and family members. This finding is similar to Teo et al. [22], in which the participants took the vaccination to protect their loved ones and to prevent the spread of influenza to their family and friends.

Despite the benefits of InVa, our participants were conflicted in their decision-making to vaccinate. Concerns about the possible side effects and effectiveness of the vaccine were predominant barriers to vaccine uptake. Such mistrust and suspicion towards the quality and effectiveness of vaccines are not new and have been cited in a number of studies [3,18,19,22,24]. Media reports of the temporary suspension of InVa in another country could have intensified our participants’ fear of vaccination, and therefore, it is imperative that we caution against the use of mass media as the only information source as the knowledge derived from it might not be entirely accurate [24]. Given that our participants’ preferred sources of information were the HCPs, followed by television or radio, it is essential that our HCPs listen to the older adults’ concerns and dispel misinformation to boost their confidence and trust in InVa.

Vaccine cost was another major economic consideration in our participants’ decision to vaccinate. Our study revealed that even though half of the participants were willing to pay for the vaccination, the majority were only willing to vaccinate if they did not need to pay out of pocket, and the cost is fully covered under MediSave. As most of our participants were currently not working, they had to rely on either their own savings or external sources of financial support. Vaccinations that require them to co-pay or self-finance would have increased their perceived financial burden, which has been associated with lower vaccination rates [17,21,28]. To cushion the impact of vaccination costs, HCPs have an important role in advising on the available subsidies, such as the Community Health Assistant Scheme for lower and middle-income individuals and utilising MediSave to pay.

Most participants in our study had no formal education or only primary education. Lower levels of education have been correlated with learning less about influenza and the need for vaccination [35]. Approximately a quarter of our participants who were unvaccinated had not heard of InVa. For the rest who had some knowledge and understanding, it seemed that knowledge insufficiency was evident. For example, our participants presumed that if they did not engage in social activities, leave their homes or travel overseas, their risk of infection was lower, and hence, they did not need to vaccinate. It is also possible that since our study was conducted during the peak of the COVID-19 pandemic, the rapid implementation of interventions, such as mask-wearing and physical distancing, could have mitigated the spread of influenza and further downplayed the need to vaccinate. There was also the existence of a “one jab is enough” mentality among our participants and hence, their belief that the COVID-19 vaccination they received could offer extended protection against influenza, given that the symptoms of both infections are similar.

To address this knowledge insufficiency, community nurses should continue to actively promote preventive health and organise community engagement programmes, such as health talks to address the potential severity of influenza, especially among those with underlying co-morbidities, and the importance of vaccine uptake in reducing the risk of influenza infection, transmission, and disease complications. These health talks should be conducted regularly and made accessible to older adults to overcome transportation inconveniences.

### Study Limitations

Data were only collected from participants who resided in the Southeast region of Singapore, spoke English, were typical of low socio-economic status and had a basic knowledge of InVa. This might limit the generalisability of the survey results and interview findings to all older adults. Secondly, our initial plan for focus groups shifted to face-to-face and telephone interviews which might narrow the scope of our interview discussion arising from the individual interviews and the lack of focus group interaction. Thirdly, we conducted telephone interviews which might limit the interpersonal connections between the interviewer and the participant. Telephone interviews might also limit the interviewer’s ability to observe the participant’s behaviour and body language.

Future studies could consider expanding participant recruitment to include non-English speaking older adults and those of diverse socio-economic statuses to increase sample representativeness and gain broader insights. Subsequent studies may also consider face-to-face focus groups to encourage participants to build on each other’s responses or recall experiences in greater detail.

## 6. Conclusions

Multifactorial influences underpinned our community-dwelling older adults’ decision to vaccinate. In light of this, multifaceted interventions will likely be more successful than single interventions in enhancing the motivators and managing the barriers. Greater community and public health efforts are needed to reach out to the larger population of community-dwelling older adults with various living arrangements. It is essential to educate older adults with knowledge insufficiency about InVa and those who were concerned about the vaccination cost, possible side effects and effectiveness of InVa. HCPs across settings need to provide more targeted information to address these concerns, especially during COVID-19, to encourage vaccine uptake.

## Figures and Tables

**Table 1:** Descriptive demographics and their association with vaccination status (N = 235).

Variables	Total Sample (N = 235)	Vaccinated (N = 93)	Non-Vaccinated (N = 114)	Unsure (N = 28)	*p*-Value
**Mean Age (SD)**	77.75 (7.28)	78.96 (7.69)	76.69 (6.60)	78.07 (8.14)	0.09
	**N (%)**				
**Gender**					0.18
Male	101 (43.0)	43 (46.2)	44 (38.6)	14 (50.0)	
Female	134 (57.0)	50 (53.8)	70 (61.4)	14 (50.0)	
**Ethnicity**					0.64
Chinese	161 (68.5)	71 (76.3)	73 (64.0)	17 (60.7)	
Malay	42 (17.9)	11 (11.8)	24 (21.1)	7 (25.0)	
Indian	28 (11.9)	10 (10.8)	15 (13.2)	3 (10.7)	
Others	4 (1.7)	1 (1.1)	2 (1.8)	1 (3.6)	
**Household type**					
1 and 2-room HDB	165 (70.2)	63 (67.7)	79 (69.3)	23 (82.1)	0.36
3/4/5-room HDB and private housing	70 (29.8)	30 (32.3)	35 (30.7)	5 (17.9)	
**Education level**					0.96
Primary and below	166 (70.6)	67 (72.0)	80 (70.2)	19 (67.9)	
Secondary and above	69 (29.4)	26 (28.0)	34 (29.8)	9 (32.1)	
**Currently working**					0.5
Yes	19 (8.1)	10 (10.8)	7 (6.1)	2 (7.1)	
No	216 (91.9)	83 (89.2)	107 (93.9)	26 (92.9)	
**Living arrangement**					0.03 *
Living alone	78 (33.2)	40 (43.0)	28 (24.6)	10 (35.7)	
Living with others	157 (66.8)	53 (57.0)	86 (75.4)	18 (64.3)	
**No. of comorbidities**					0.51
1	116 (49.4)	43 (46.2)	57 (50.0)	16 (57.1)	
2	82 (34.9)	32 (34.4)	44 (38.6)	6 (21.4)	
3 or more	37 (15.7)	18 (19.4)	13 (11.4)	6 (21.5)	
**Have regular family doctor or polyclinic**					0.96
Yes	221 (94.0)	90 (96.8)	106 (93.0)	25 (89.3)	
No	14 (6.0)	3 (3.2)	8 (7.0)	3 (10.7)	
**Live near primary care facilities**					0.58
Yes	227 (96.6)	89 (95.7)	110 (96.5)	28 (100)	
No	8 (3.4)	4 (4.3)	4 (3.5)	-	
**Travel**					0.14
Less than once a year	196 (83.4)	82 (88.2)	90 (78.9)	24 (85.7)	
At least once a year	39 (16.6)	11 (11.8)	21 (21.1)	4 (14.3)	

Note: SD = Standard Deviation; HDB = Housing and Development Board flat; * indicates *p* < 0.05.

**Table 2:** Multinomial logistic regression comparing vaccinated (N = 93) and non-vaccinated (N = 114).

Variable	Vaccinated (N= 93)	
	Sig.	Odds Ratio (95% CI)
**Age**	0.067	1.041 (0.997–1.087)
**Gender**		
Male	0.2	1.495 (0.809–2.764)
Female	-	-
**Household type**		
	0.195	0.628 (0.311–1.269)
1 and 2-room HDB	-	-
3/ 4/ 5-room HDB and private housing		
**Education level**		
	0.592	0.822 (0.402–1.682)
Primary and below	-	-
Secondary and above		
**Currently working**		
	0.066	0.328 (0.100–1.076)
No	-	-
Yes		
**Living arrangement**		
Living alone	0.006 *	2.504 (1.294–4.842)
Living with others	-	-
**No. of comorbidities**		
1	0.106	0.487 (0.204–1.164)
2	0.133	0.494 (0.197–1.239)
3 or more	-	-
**Have regular family doctor or polyclinic**		
No	0.208	0.373 (0.080–1.732)
Yes	-	-
**Live near primary care facilities**		
No	0.869	0.883 (0.199–3.913)
Yes	-	-
**Travel**		
Less than once a year	0.068	2.357 (0.939–5.916)
At least once a year	-	-

Note: Reference category: Non-vaccinated (N = 114); HDB = Housing and Development Board flat; * indicates *p* < 0.05.

**Table 3:** Attitudes towards influenza infections and vaccinations (N = 235).

Statement	Strongly Agree (%)	Agree (%)	Neutral (%)	Disagree (%)	Strongly Disagree (%)
I feel that influenza is a potentially serious illness.	35.7	37.0	17.0	9.8	0.4
I feel that I am at risk of getting an influenza infection.	23.8	23.4	20.0	30.2	2.6
I feel that it is important to get myself vaccinated against influenza.	36.2	37.4	12.8	12.3	1.3
I feel that regular influenza vaccination is important for ALL adults aged 65 years and older.	35.3	36.6	17.4	10.2	0.4
I feel that regular influenza vaccination is important for persons with chronic medical diseases.	37.0	34.5	18.7	9.4	0.4
I feel that it is only important to get influenza vaccination before an overseas trip.	31.9	36.2	15.7	13.6	2.6

**Table 4:** Intention for future vaccination (N = 235).

Reasons/Situations	Not Important at all (%)	Slightly Important (%)	Fairly Important (%)	Important (%)	Very Important (%)
**Enablers**					
Avoidance of getting infected	2.1	4.3	11.1	28.9	53.6
Avoidance of transmission to others	1.7	3.4	10.2	34.9	49.8
Being advised by a healthcare professional to receive the vaccination	1.7	6.4	8.5	32.3	51.1
Being encouraged by my family and/or friends to receive the vaccination	7.7	9.8	19.6	27.7	35.3
Having vaccination services conveniently available nearby	3.8	6.4	14.0	26.0	49.8
Having vaccination events organised together with other activities e.g., health screening	9.4	6.0	14.5	28.5	41.7
**Barriers**					
Concerns about possible side effects	23.0	15.7	20.0	19.1	22.1
Concerns about effectiveness	20.4	14.0	23.0	24.3	18.3
Preference to use other protective measures e.g. complementary alternative medicine	25.1	19.6	26.4	17.0	11.9
Not having enough information	13.6	11.1	27.2	23.4	24.7

**Table 5:** Willingness-to-pay (N = 235).

Questions	Very Willing (%)	Somewhat Willing (%)	Not Willing (%)
How willing are you to pay for the influenza vaccination at SGD 35 per dose?	32.8	23.0	44.3
How willing are you to be vaccinated if the cost is fully covered under MediSave?	75.7	11.9	12.3

**Table 6:** Preferred vaccination sites and sources of information (Multiple choices allowed).

Variables	Frequency (N)	Percentage (%)
**Vaccination sites**		
Polyclinic	130	55.3
Community spaces (e.g. community centre, senior activity centre)	92	39.1
General practitioner/family clinic	51	21.7
Public hospital	37	15.7
Others (e.g. home)	19	8.1
No preference	15	6.4
Not keen for vaccination	15	6.4
Private hospital	1	0.4
**Sources of Information**		
Healthcare professional	171	72.8
Television/radio	73	31.1
Roadshow	45	19.1
Family	35	14.9
Printed materials	31	13.2
Friend	25	10.6
Others (i.e. community centre, senior activity centre)	19	8.1
Social media	11	4.7
Website	10	4.3

**Table 7:** Interview participants (N = 20).

Participant Code	Age	Gender	Household Type	Living Alone	Vaccination Status	Number of Comorbidities
P1	77	Female	2-room	Yes	Yes	2
P2	71	Female	2-room	No	No	2
P3	82	Male	3-room	Yes	No	2
P4	77	Female	2-room	Yes	No	4
P5	80	Female	1-room	Yes	Yes	1
P6	75	Male	2-room	Yes	Yes	3
P7	73	Male	1-room	No	No	0
P8	74	Male	1-room	No	No	1
P9	82	Female	1-room	Yes	Yes	1
P10	71	Female	1-room	No	No	1
P11	69	Female	3-room	Yes	Yes	1
P12	71	Female	3-room	Yes	Yes	2
P13	71	Female	1-room	Yes	Yes	2
P14	73	Male	1-room	Yes	Yes	2
P15	77	Male	2-room	Yes	No	2
P16	68	Female	3-room	Yes	No	1
P17	75	Female	5-room	No	No	1
P18	72	Female	4-room	No	No	1
P19	66	Female	3-room	No	No	1
P20	73	Male	2-room	No	No	2

## Data Availability

The data that support the findings of this study are available from the corresponding author, Xu Yi, upon reasonable request.

## References

[1] Mereckiene J., Cotter S., Nicoll A., Lopalco P., Noori T., Weber J.T., D’Ancona F., Lévy-Bruhl D., Dematte L., Giambi C. (2014). Seasonal influenza immunization in Europe. Overview of recommendations and vaccination coverage for three seasons: Pre-pandemic (2008/09), pandemic (2009/10), and post-pandemic (2010/11). Eurosurveillance.

[2] Worasathit R., Wattana W., Okanurak K., Songthap A., Dhitavat J., Pitisuttithum P. (2015). Health education and factors influencing acceptance of and willingness to pay for influenza vaccination among older adults. BMC Geriatr..

[3] Gallant A.J., Flowers P., Deakin K., Cogan N., Rasmussen S., Young D., Williams L. (2021). Barriers and enablers to influenza vaccination uptake in adults with chronic respiratory conditions: Applying the behaviour change wheel to specify multi-levelled tailored intervention content. Psychol. Health.

[4] World Health Organization Influenza (Seasonal). https://www.who.int/news-room/fact-sheets/detail/influenza-.

[5] Chan D.P., Wong N.S., Wong E.L., Cheung A.W., Lee S.S. (2015). Household characteristics and influenza vaccination uptake in the community-dwelling elderly: A cross-sectional study. Prev. Med. Rep..

[6] Center for Disease Control and Prevention [CDC]: Study Shows Hospitalization Rates and Risk of Death from Seasonal Flu Increase with Age among People 65 Years and Older. https://www.cdc.gov/flu/spotlights/2018-2019/hopitalization-rates-older.html.

[7] Ang L.W., Lim C., Lee V.J.M., Ma S., Tiong W.W., Ooi P.L., Lin R.T.P., James L., Cutter J. (2014). Influenza-Associated Hospitalizations, Singapore, 2004–2008 and 2010–2012. Emerg. Infect. Dis..

[8] World Health Organization Seasonal Influenza Vaccines: An Overview for Decision-Makers. https://apps.who.int/iris/handle/10665/336951.

[9] Chung J.R., Rolfes M.A., Flannery B., Prasad P., O’Halloran A., Garg S., Fry A.M., Singleton J.A., Patel M., Reed C. (2020). Effects of Influenza Vaccination in the United States During the 2018–2019 Influenza Season. Clin. Infect. Dis..

[10] Kwong E., Pang S.M.-C., Choi P.-P., Wong T.K.-S. (2010). Influenza vaccine preference and uptake among older people in nine countries. J. Adv. Nurs..

[11] McIntyre A., Zecevic A., Diachun L. (2014). Influenza vaccinations: Older adults’ decision-making process. Can. J. Aging.

[12] Yeung M.P., Ng S.K.-C., Tong E.T.F., Chan S.S.-K., Coker R. (2015). Factors associated with uptake of influenza vaccine in people aged 50 to 64 years in Hong Kong: A case–control study. BMC Public Health.

[13] Young B.E., Chen M. (2020). Influenza in temperate and tropical Asia: A review of epidemiology and vaccinology. Hum. Vaccines Immunother..

[14] Yue M., Wang Y., Low C.K., Yoong J.S.-Y., Cook A.R. (2020). Optimal Design of Population-Level Financial Incentives of Influenza Vaccination for the Elderly. Value Health.

[15] Ministry of Health Singapore [MOH]: MOH Establishes National Adult Immunization Schedule; Extends Use of MediSave for Vaccines under the Schedule. https://www.moh.gov.sg/news-highlights/details/moh-establishes-national-adult-immunisation-schedule-extends-use-of-medisave-for-vaccines-under-the-schedule.

[16] Jain A., van Hoek A., Boccia D., Thomas S.L. (2017). Lower vaccine uptake amongst older individuals living alone: A systematic review and meta-analysis of social determinants of vaccine uptake. Vaccine.

[17] Sun K.S., Lam T.P., Kwok K.W., Lam K.F., Wu D., Ho P.L. (2020). Seasonal influenza vaccine uptake among Chinese in Hong Kong: Barriers, enablers and vaccination rates. Hum. Vaccines Immunother..

[18] Chen C.-H., Wu M.-S., Hsu W.-Y., Chen Y.-M., Hsu C.-C., Hsiung C.A., Wu I.-C. (2017). Determinants of influenza vaccination in older adults: A nationwide community-based study in Taiwan. Geriatr. Gerontol. Int..

[19] Yan S., Wang Y., Zhu W., Zhang L., Gu H., Liu D., Zhu A., Xu H., Hao L., Ye C. (2021). Barriers to influenza vaccination among different populations in Shanghai. Hum. Vaccines Immunother..

[20] Tan E.K., Lim L.H., Teoh Y.L., Ong G., Bock H.L. (2010). Influenza and seasonal influenza vaccination among diabetics in Singapore: Knowledge, attitudes and practices. Singap. Med. J..

[21] Ang L.W., Cutter J., James L., Goh K.T. (2017). Factors associated with influenza vaccine uptake in older adults living in the community in Singapore. Epidemiol. Infect..

[22] Teo L., Smith H., Lwin M., Tang W. (2019). Attitudes and perception of influenza vaccines among older people in Singapore: A qualitative study. Vaccine.

[23] Panchapakesan C., Sheldenkar A., Cayabyab Y.M., Ng J.S., Lu J., Lwin M.O. (2018). A Comparison between the Predictors of Vaccine Uptake Intentions for Influenza and Dengue. Int. J. Environ. Res. Public Health.

[24] Kan T., Zhang J. (2018). Factors influencing seasonal influenza vaccination behaviour among elderly people: A systematic review. Public Health.

[25] Fusch P.I., Ness L.R. (2015). Are We There Yet? Data Saturation in Qualitative Research. Qual. Rep..

[26] Braun V., Clarke V. (2006). Using thematic analysis in psychology. Qual. Res. Psychol..

[27] Laerd Statistics. Multinomial Logistic Regression Using SPSS Statistics. https://statistics.laerd.com/spss-tutorials/multinomial-logistic-regression-using-spss-statistics.php.

[28] Bazargan M., Martinez-Hollingsworth A., Cobb S., Kibe L.W. (2022). Correlates of influenza vaccination among underserved Latinx middle-aged and older adults: A cross-sectional survey. BMC Public Health.

[29] Xu Y., Koh X.H., Chua Y.T.S., Tan C.G.I., Aloweni F.A.B., Yap B.E.J., Tan P.C., Chua X., Lim Y.K.S., Oh H.C. (2022). The impact of community nursing program on healthcare utilization: A program evaluation. Geriatr. Nurs..

[30] Cummings C.L., Kong W.Y., Orminski J. (2020). A typology of beliefs and misperceptions about the influenza disease and vaccine among older adults in Singapore. PLoS ONE.

[31] Li Q., Zhang M., Chen H., Wu F., Xian J., Zheng L., Liang M., Cao H., Zhou X., Gu Z. (2021). Influenza Vaccination Coverage among Older Adults with Hypertension in Shenzhen, China: A Cross-Sectional Survey during the COVID-19 Pandemic. Vaccines.

[32] Barry M.A., Aljammaz K.I., Alrashed A.A. (2020). Knowledge, Attitude, and Barriers Influencing Seasonal Influenza Vaccination Uptake. Can. J. Infect. Dis. Med. Microbiol..

[33] Okoli G.N., Lam O.L.T., Racovitan F., Reddy V.K., Righolt C.H., Neilson C., Chit A., Thommes E., Abou-Setta A.M., Mahmud S.M. (2020). Seasonal influenza vaccination in older people: A systematic review and meta-analysis of the determining factors. PLoS ONE.

[34] Rosenstock I.M., Glanz K., Lewis F.M., Rimer B.K. (1990). The health belief model: Explaining health behaviour through expectancies. Health Behaviour and Health Education: Theory, Research, and Practice.

[35] Khoury G.E., Salameh P. (2015). Influenza vaccination: A cross-sectional survey of knowledge, attitude, and practices among the Lebanese adult population. Int. J. Environ. Res. Public Health.

